# Coaching leaders toward favorable trajectories of burnout and engagement

**DOI:** 10.3389/fpsyg.2023.1259672

**Published:** 2023-12-04

**Authors:** Pilar Jasmine Brooks, Pilar Ripoll, Carmen Sánchez, Marta Torres

**Affiliations:** ^1^Institut d'Investigació en Psicologia dels Recursos Humans, del Desenvolupament Organitzacional, i de la Qualitat de Vida Laboral (Idocal), Universitat de València, Valencia, Spain; ^2^INTELEMA, Valencia, Spain

**Keywords:** executive coaching, burnout, engagement, leadership intervention, psychological well-being, quality of work life

## Abstract

Existing literature on executive coaching has shown beneficial outcomes for leaders. Indeed, executive coaching can positively affect an individual’s psychological well-being and quality of work life. However, while burnout among leaders is on the rise, to our knowledge no prior study has investigated the influence of executive coaching on the dimensions of burnout and engagement concurrently. Therefore, the aim of the current study is to examine if an executive coaching intervention has positive effects on managerial burnout and engagement. We implemented a ten-week coaching intervention for leaders, where questionnaire data were collected at two time points. Participants (*N* = 92; *M*_age_ = 42; 64% male) were randomly assigned to an intervention or a wait-list control group. Self-reported levels of engagement and burnout were collected at the start of the study protocol (T1) and at the end (T2). Coaching sessions for the intervention group were one hour once per week. The control group did not receive coaching. Repeated measures ANOVAs examined the extent to which the intervention influences the leader’s engagement and burnout. Analyses revealed that all three dimensions of burnout significantly decreased for the intervention group over time while vigor increased. Findings did not reveal an increase in dedication and absorption. Consistent with prior literature, this study supports that executive coaching can improve a leader’s well-being. Specifically, this study shows that an executive coaching intervention can decrease burnout symptoms and increase vigor in leaders. Adding a follow-up survey to the design would allow for further exploration of the intervention on engagement.

## 1 Introduction

Reports of employee burnout are increasing every year globally (Gallup Inc, 2021; Future Forum, 2022). In fact, in 2022 the Future Forum Pulse surveyed over 10,000 workers and found that even leaders are reporting higher rates of burnout, from middle managers at 43% to executives at 32%. In addition, the survey results also revealed that individuals who indicated they were burnt out were 22 times more likely to report higher stress and anxiety levels. Consequently, individuals experiencing elevated rates of stress and burnout tend to also have lower levels of physical well-being and work-life balance (Gallup Inc, 2021). Thus, for leaders, these negative consequences likely hinder positive management practices. Indeed, previous research has shown that leaders who experience burnout enact lower managerial quality (Parent-Lamarche and Biron, 2022) and that stress can influence a leader’s behavior; in turn influencing employee stress (Skakon et al., 2010; Harms et al., 2017). Given the current rise in entrepreneurial burnout (Global Leadership Forecast, 2021 as cited by Arora et al., 2023), this is particularly concerning provided that when leaders burnout the resulting negative outcomes can act as a contagion that trickles down the organization’s hierarchy (Maslach et al., 2001; Schippers and Hogenes, 2011). Thus, burnout among leaders not only negatively impacts their own well-being and work performance, but can also have adverse effects on the employee. Therefore, it is pertinent to seek ways in which to reduce factors leading to burnout in leaders. Furthermore, while burnout is characterized by lower well-being and work performance, engagement is the antithesis with opposing characteristics such as increased well-being and favorable workplace outcomes (Bakker et al., 2014). Thus, it is equally important that interventions focused on reducing burnout levels in leaders also seek to concurrently improve their levels of engagement. In this instance, it could be useful to look toward forms of leadership development such as learning practices that aim to aid a leader’s individual development while supporting their goals within the organizational context. Organizational learning of this nature often takes place in a one-to-one conversation with an outside professional such as a coach (de Haan et al., 2020). This leader and coach relationship is known as executive coaching and it often functions to improve the leader’s self-awareness, learning, and occupational success (Joo, 2005).

Consequently, prior research suggests that executive coaching is one such tool that may prove to be an effective combatant for factors related to burnout by improving their workplace well-being (Grant et al., 2009). Research has also suggested that executive coaching can improve leaders’ personal engagement in their work by way of enabling them to feel more productive and satisfied in the workplace (Kombarakaran et al., 2008). However, research on executive coaching interventions is considerably lacking when accounting for its popularity in practice (Grover and Furnham, 2016). Additionally, there is only a small amount of research that has utilized controlled interventions for examining burnout (Maricuţoiu et al., 2016). Thus, researchers are less able to determine if any resulting effects are indeed from the intervention process or if results were influenced by an extraneous source. Moreover, many studies examining the outcomes of coaching procedures have not used longitudinal study designs (Grover and Furnham, 2016). Further, studies examining how coaching may influence burnout or engagement is limited (Grover and Furnham, 2016; de Haan and Nilsson, 2023). Thus, to the best of our knowledge, there has been no research examining both burnout and engagement in business leaders. Therefore, the present study sought to examine the potential longitudinal effects on managerial burnout and engagement after implementing an executive coaching procedure. Further, we aimed to address the gaps in the literature by using a randomized control trial design to allow for a more meaningful understanding of any intervention effects on leader’s burnout and engagement levels.

## 2 Theoretical background

### 2.1 Executive coaching

Coaching is a form of intervention that seeks to facilitate an individual’s learning about their unrealized ability to enhance their own performance (Whitmore, 1992). Specifically, coaching has been typically used to intervene in the development and performance of employees, healthcare workers, athletes, and teachers (Bagi, 2013). Additionally, it is often a leader that acts as the facilitator of the coaching process (Carvalho et al., 2021). However, the field of coaching has begun to branch out into more specialized forms of interventions, such as coaching outside of the workplace for improving health (i.e., health coaching) or an individual’s life (i.e., life coaching) (Passmore and Lai, 2019). Additionally, another occupational subtype emerged around the end of the 1980’s, where coaching is focused at the leader level and is referred to as executive coaching (Tobias, 1996). Furthermore, research has revealed some agreement regarding the main aspects of coaching (Jones et al., 2016). In executive coaching, a leader within an organization and a coach develop and maintain a relationship where the coach facilitates the creation of mutually agreed upon clearly defined goals for the leader (Grant et al., 2009; Smither, 2011; Jones et al., 2016). Thus, executive coaching is a goal-oriented process (Grant, 2006). Additionally, executive coaching is also solution-focused, as the coaching process aims to review a leader’s inter/intrapersonal difficulties in order to identify and implement effective tools and methods for the production of positive workplace outcomes. Furthermore, executive coaching is considered to have a broader positive impact than other forms of individualized coaching given that the benefits the leader reaps can seep down into the organization as well (Ellam-Dyson et al., 2019). Thus, in this vein of thinking, executive coaching can also be considered a top-down approach to addressing some organizational problems. However, literature on how best to logistically implement executive coaching in practice is limited. For example, research on the prescribed amount and frequency of executive coaching is mixed (Sonesh et al., 2015; Pandolfi, 2020). Thus, more experimental research is warranted to expand the breadth of understanding as to what variances in executive coaching designs account for differing outcomes for coachees.

#### 2.1.1 The GROW model

To guide our intervention development, we used a behavioral-based coaching model called GROW. The GROW model—often associated with Sir John Whitmore who is known as one of the founders of coaching—was originally developed by Graham Alexander (Passmore, 2019). Additionally, research has revealed that GROW is one of the most commonly used coaching models today (Lai and Palmer, 2019). Likely, this is due in part to the model being so robust and flexible (Panchal and Riddell, 2020). Further, GROW is very accessible in that it can be used by coaches with no former training in psychology (Passmore, 2019). Specifically, GROW is a four-stage model rooted in behaviorism that involves a coach offering open-ended questions to facilitate a coachee in determining the behaviors needed to achieve specified goals. In Passmore’s (2019) “Behavioral Coaching” chapter in the *Handbook for Coaching Psychology,* he provided a detailed outline of the four stages of GROW. Here, he specifies how the stages are set up in sequential order, however, often it is necessary to return to prior stages multiple times throughout the coaching process. Thus, while GROW may appear to be a linear model it is intended to be recursive (Whitmore, 2017).

The first stage includes identifying a specific goal. It is deemed necessary to achieve goal clarity to allow the coachee to recognize their ability in achieving the stated goal. Additionally, it helps the coach to understand how they can best support the coachee. Furthermore, the goal should provide an adequate amount of challenge by which the coachee is intrinsically motivated to achieve the goal. In addition, a goal setting process should occur so that short-term goals are specified and add up to a larger long-term goal.

Next, the second stage of GROW requires the coach to aid the coachee in realizing their current reality. Thus, allowing the coachee to discover how their goals fit into their reality. The process by which a coachee explores their reality is often self-reflective. Additionally, a coach may choose to administer a 360-degree questionnaire (e.g., a skills assessment completed by subordinates, peers, supervisors, and the individual of interest) to utilize the resulting data as a point of analysis and reflection in the coaching process. Furthermore, the examination of the outcomes from the self-reflection and 360-degree assessments can help the coachee to recognize whether their goals are realistic.

The third stage is intended to initiate the coachee in determining what options they have available to them for achieving their goals. Here, the options are critically analyzed and sometimes weighted against one another to ensure the options chosen are appropriate as well as doable. Finally, the fourth stage has the coachee state an action plan for accomplishing their goals. Additionally, the coachee proposes a timeline in which the coach and coachee can review the progression of the goal achievement process. In the coaching sessions to follow the coach and coachee will engage in a detailed examination of the progress where setbacks and accomplishments will be addressed. The conclusions drawn will allow the coach to facilitate the coachee in continuing their path toward goal achievement by way of highlighting those behaviors that appear to elicit a positive direction toward the coachee’s specific goals.

To conclude, Passmore (2019) identifies that leaders are likely to benefit greatly from the GROW model given that, often, they already have goals that they are working to attain. Thus, GROW appears to be a useful executive coaching tool to foster and promote a leader’s motivation toward their work, while acknowledging and mitigating behaviors that are not serving their purpose. Therefore, it is likely that coaching interventions that utilize the GROW framework can address methods for reducing burnout and increasing work engagement.

#### 2.1.2 Outcomes of executive coaching

Similar to coaching for employees, executive coaching is a form of intervention that can help improve a leader’s well-being and work performance (Grant et al., 2009; Theeboom et al., 2014; Jones et al., 2016; Passmore and Lai, 2019). Moreover, executive coaching has been linked with many positive outcomes. Notably, a systematic-analysis by Athanasopoulou and Dopson’s (2018) revealed over 70 positive outcomes from executive coaching. Here, some of the outcomes identified included those that benefited the leader personally (i.e., less stress, increased life satisfaction, more resiliency, and improved time-management), those that benefited the relationships leaders had with others (i.e., improved management and communication skills), those that reflected the interaction between the leader and their work (i.e., increased work performance, more felt relatedness to the organization, and feelings of value at work), and those that created benefits at the organizational level (i.e., higher satisfaction among employees, increased productivity, and more effective leadership). Notably, since the publication of this meta-analysis, further studies on executive coaching have contributed to supporting the findings just mentioned and adding new positive outcomes in the process. For example, in a study that implemented an executive coaching program ending in 2020, the leaders who participated in the program reported that they felt more hope and resiliency when faced with the COVID-19 pandemic (Addison and Shapiro, 2023). Indeed, executive coaching may have been a useful resource for leaders in the face of such uncertainty like the pandemic. For example, in a study that was conducted during the pandemic, leaders who participated in executive coaching sessions reported more well-being and better work performance when compared to those who did not attend coaching sessions (Jarosz, 2021). Thus, the positive effects of executive coaching appear to be extensive in that the leader, the organization, and the employees can all profit.

### 2.2 Burnout

“Burnout is a psychological syndrome of emotional exhaustion, depersonalization, and reduced personal accomplishment that can occur among individuals who work with other people in some capacity” (Maslach et al., 1996, p. 192). However, current research has moved toward a more general understanding of burnout through three specific dimensions: emotional exhaustion (e.g., perceived depletion of mental and physical energy at work), cynicism (e.g., indifferent attitude toward work and members in the workplace), and professional inefficacy (e.g., amount one perceives they are unable or incompetent to perform work tasks) (Maslach and Jackson, 1981; Edú-Valsania et al., 2022; World Health Organization, 2022). Emotional exhaustion, cynicism, and professional inefficacy are all symptoms that occur over time and often in response to aspects of an individual’s work environment (Maslach and Leiter, 2008). Taken together, burnout is a process by which a person gradually loses enthusiasm and vigor. Also of note, burnout symptoms can range on a continuum (Bakker et al., 2014). Here, at the highest level, burnout symptoms occur over an extended period of time and often an individual requires a long recovery period (Schaufeli et al., 2009). Consequently, high levels of burnout have been associated with depression (Toker and Biron, 2012), social contact avoidance (Casserley and Megginson, 2009), suicidal ideation (Dyrbye et al., 2008), and less satisfaction with life (Hakanen and Schaufeli, 2012). More specifically, within the work context, burnout symptoms can manifest into feelings of exploitation (Bakker and de Vries, 2021), loss of dedication to their work and job dissatisfaction (Maslach and Leiter, 2008), as well as low levels of job performance (Demerouti et al., 2014). Thus, constant workplace stressors may develop into severe outcomes for individuals. For instance, in the leader’s case, occupational distress negatively impacts a leader’s ability to support employees (Skakon et al., 2010) and find work-life balance (Bunea, 2020).

To illustrate, some indicators linked to these undesirable outcomes include the accumulation of feeling drained, tired, and continuously fatigued; in other words individual’s are exhausted (Maslach et al., 2001). More specifically, an individual who experiences exhaustion from a depletion of emotional resources expended in the workplace, is said to be emotionally exhausted (Roche and Haar, 2013). Interestingly, emotional exhaustion can occur when an individual is faced with incongruent workplace demands and perceives little resources available to cope. For example, when leaders are unable to use personal resources, such as quickly displaying positive emotions when confronted with negative emotionally charged events (i.e., display emotional resiliency), they are more likely to burnout (Casserley and Megginson, 2009; Garrosa and Moreno-Jiménez, 2013). Furthermore, leadership styles used by a leader, such as transformational leadership, can deplete a leader’s personal resources and as a result increase their emotional exhaustion (Zwingmann et al., 2016).

Consequently, individuals may cope with emotional exhaustion by detaching themselves from the source of the drain (Bolton et al., 2012). However, while psychological detachment has been shown to buffer the positive relationship between work stress and emotional exhaustion (Sonnentag et al., 2010), many individuals detach from members in the work place instead (Parayitam et al., 2022). Thus, a leader who is unable to connect with their employees and other members of management will likely find it difficult to find organizational success. Further, it should be noted that detaching oneself from other members of the workplace is a common coping mechanism known as depersonalization. Interestingly, depersonalization commonly goes along with an individual formulating an enduring negative assessment about various aspects of their work place (e.g., other employees, the organization, their job) (Maslach et al., 2001). Accordingly, an individual is said to have become cynical. Individuals who display cynicism tend to do so when they perceive a lack of job resources (e.g., job control, feedback from their work, and ability to contribute to decision making) (Demerouti et al., 2001). Ultimately, individuals displaying cynicism no longer are interested in their job or derive meaning from their work (Maslach et al., 2001). Thus, when both emotional exhaustion and cynicism are present in a person, it is likely that they also experience feelings of being incompetent or unsuccessful in their work endeavors (Maslach and Leiter, 2008). Such feelings of failure are what encompass the component of burnout known as personal inefficacy. Personal inefficacy is of particular concern when present in leaders as the symptom can result in organizational problems. To illustrate, one of the key components of leadership is to inspire followers and when a leader lacks personal efficacy they are unable to inspire themselves at work, let alone inspire others. Also of note, high achieving employees are not always reliable in perceiving their progression toward burnout (Casserley and Megginson, 2009). Thus, efforts to prevent burnout symptoms from arising in leaders is crucial for preventing organizational dysfunction.

Therefore, designing effective interventions to address the increasing indices of burnout among leaders is becoming ever more imperative. Thus far, prior literature has proposed various suggestions toward preventative and mitigative processes (Schaufeli and Enzmann, 1998; Schaufeli and Bakker, 2004; Bagi, 2013; Maricuţoiu et al., 2016; Haar et al., 2018; Gabriel and Aguinis, 2022; Bachman et al., 2023). Indeed, such suggestions include leader’s taking extended periods of time off from work, seeking support from people within their personal and work life, and creating changes in their identity or lifestyle (Bagi, 2013). Additionally, interventions that help to develop a leader’s interpersonal and role-related skills have also been suggested (Maslach et al., 2001; Maricuţoiu et al., 2016). Furthermore, personal interventions initiated by the leader themselves have been proposed (Bachman et al., 2023). Self-initiated interventions in this manner may include implementing time management strategies to ease job demands (Li et al., 2018). Moreover, Schaufeli and Bakker (2004) highlighted the importance of other individual-based interventions (e.g., self-monitoring, self-awareness, creating healthy lifestyles, relaxation, and cognitive behavioral techniques; Schaufeli and Enzmann, 1998). Currently, popular interventions for reducing burnout include those related to stress management such as cognitive-behavioral therapy (i.e., a form of problem-oriented psychotherapy) and relaxation interventions (i.e., mindfulness meditation; Haar et al., 2018; Gabriel and Aguinis, 2022). However, research has found that while both cognitive-behavioral therapy and relaxation interventions can help to reduce emotional exhaustion, they do not reduce other dimensions of burnout (Maricuţoiu et al., 2016). Thus, effective interventions that can help reduce all dimensions of burnout require further consideration and examination.

### 2.3 Engagement

Prior research has pointed to burnout as an erosion of work engagement, where burnout is considered the polar opposite of engagement (Afrahi et al., 2022). More specifically, “Schaufeli and his colleagues have defined engagement as a persistent, positive affective-motivational state of fulfillment in employees that is characterized by vigor, dedication, and absorption” (Maslach et al., 2001, p. 417). Here, vigor is understood as an individual’s invested, energetic, resilient work ethic including perseverance when faced with challenges, dedication is depicted when an individual is deeply connected to one’s work so much that they feel challenged, eager and a sense of meaning, and absorption is characterized as feeling joyful whilst fully attending to one’s work (Schaufeli et al., 2002; Maslach and Leiter, 2008; Bakker et al., 2014). Thus, the three dimensions cumulate to an individual feeling that their work is fulfilling (Schaufeli et al., 2002). Indeed, engagement has also been characterized by an individual’s perception of responsibility and commitment to their job (Britt et al., 2001). Moreover, work engagement has been conceptualized as a global attitude an individual has toward their work. Accordingly, engagement has been linked with increased work performance (Mackay et al., 2016). Additionally, engagement may increase psychological well-being by the elicitation of positive emotions (Shirom, 2007). Thus, when an individual is engaged, they may feel happier which in turn improves their mental health.

However, work engagement appears to be more closely related to motivational outcomes rather than health-related outcomes (Bakker et al., 2014). Indeed, prior research has attributed work engagement as a product of an individual fulfilling their psychological needs (e.g., relatedness, competence, and autonomy) within the workplace (Nimon and Shuck, 2020). This is in line with self-determination theory where it is said that individuals are motivated by their own psychological needs: being able to acquire knowledge (e.g., competency), having freedom in their choices (e.g., autonomy), and feeling as if we belong (e.g., relatedness) (Ryan and Deci, 2000). Thus, it could be that when a person is highly engaged in their work, they are being driven by their psychological needs. Consequently, it has also been proposed that when an individual becomes unmotivated they do not remain engaged in their work (e.g., disengaged) (Afrahi et al., 2022). Therefore, as mentioned previously, a disengaged worker is more likely to develop cynicism. However, it is important to note that while an individual may be disengaged this does not necessitate that they are burnt out (Schaufeli and Bakker, 2004). Though, when an individual notices they are disengaging, it could be helpful to look toward ways to increase engagement so as to prevent the potential burnout symptoms from manifesting (Bagi, 2013). Notably, having engaged leaders within an organization is important for more than just avoiding burnout symptoms. As illustrated previously, burnout symptoms can have a trickle-down effect within the organization from the leader to follower; coincidently, this same effect can be seen with engaged leaders (Lu et al., 2018; Addison and Shapiro, 2023). In other words, when a leader is engaged at work, they can act as a role model for their followers, who mimic the leader’s behavior and thus become more engaged in their work as well. Moreover, given that work engagement has been linked with more well-being, it is advantageous for organizations to seek ways in which they can increase work engagement for their leaders as to benefit not only the leader, but also the employees.

Thus far, interventions aimed at increasing work engagement have found some success. Indeed, a recent meta-analysis by Knight et al. (2019) found that mindfulness-based interventions have shown promising effects for increasing employee work engagement. However, many of the interventions included in the analyses either did not examine nor find significant positive results for each of the three dimensions of work engagement. Consequently, a more recent meta-analysis examined controlled interventions aimed at increasing engagement levels and here the researchers did find positive significant effects of the interventions on all three dimensions of engagement (Vîrgă et al., 2021). However, like Knight et al.’s (2019) review, the interventions included did not examine leaders specifically. In fact, most of the organizational literature that has investigated ways to intervene in the development of burnout and the promotion of work engagement, have focused on the employee versus the leader (Maricuţoiu et al., 2016; Knight et al., 2019; Gabriel and Aguinis, 2022). Therefore, interventions aimed at increasing engagement and decreasing burnout specifically in leaders is an area that needs more consideration.

### 2.4 Executive coaching, burnout, and engagement

Coaching has been shown to be an effective intervention to reduce negative workplace outcomes such as burnout in the early stages of the condition for employees (Grant, 2017; Edú-Valsania et al., 2022). Furthermore, research on executive coaching has revealed increases in the level of a coachee’s occupational motivation and decreases in their level of stress (Sonesh et al., 2015). Additionally, preliminary research on executive coaching has suggested that leaders who perceive they have engaged in a high quality coaching relationship can lead to improving their level of work engagement (Van Oosten et al., 2019). That is, there does appear to be a link between executive coaching and higher work engagement in leaders. However, there is very little research on how the outcomes of the coaching procedure (e.g., engagement) arise as a function of the tools used (e.g., SWOT analysis, feedback, etc.). Specifically, there is a need for further research on how certain executive coaching methods work (Pandolfi, 2020). Notwithstanding, the tools selected are often based on the particular coaching method applied. Our study used a person-centered approach which is founded in the idea that individuals are inherently motivated to change their behavior because they themselves want to change (i.e., they are intrinsically motivated) (Joseph, 2003). Person-centered coaching can help guide individuals to make more authentic choices for themselves. Therefore, leaders may benefit from person-centered coaching to find more purpose in their work and therefore become more engaged. Increased work engagement is beneficial not only to the improvement of an individual’s work performance, but also to their personal well-being (Knight et al., 2019). However, despite these findings there is little research on interventions for leaders to address burnout or work engagement (Bakker et al., 2011; Grover and Furnham, 2016). Equally important, there is simply a lack of research that focuses on the well-being of leaders at all (Wirtz et al., 2017). Consequently, there is a call in the literature for coaching as a support system for leaders to avoid burnout (Bagi, 2013; Haar, 2021). In fact, a vast majority of leaders themselves have called for coaching to mitigate work-related stressors (Campbell et al., 2007). Therefore, it is pertinent that research be conducted on ways in which executive coaching can aid leaders in navigating occupational stressors.

Currently, one study has investigated how coaching may improve leader’s well-being and reduce their burnout (McGonagle et al., 2020). The researchers of this study gathered a sample (*N* = 59) of primary care physicians from the United States who were randomly assigned to an intervention group (*n* = 29, *M*_age_ = 43) and a waitlist control group (*n* = 30, *M*_age_ = 42). After completing a baseline survey, the intervention group participated in six coaching sessions approximately every two weeks over a three-month period. Here, the coaching sessions lasted 30 min over the phone apart from an initial in-person session lasting 60 min. The waitlist control group did not partake in any coaching during this three-month period. Both groups (e.g., intervention and control) completed online surveys pre-intervention, post-intervention, as well as at two follow up times post-intervention (e.g., three months and six months). Nine outcome measures were assessed in each survey, of which two outcome measures included burnout which was assessed with the Maslach Burnout Index (Maslach et al., 1996) and engagement which was assessed with a 17-item engagement scale (Rich et al., 2010). Repeated measures ANOVAs revealed a significant interaction effect of time and group on burnout and engagement. Additionally, multiple comparison tests revealed that for the intervention group, burnout decreased from pre-intervention to post-intervention while engagement increased during this same period. Thus, this study suggests that executive coaching can be an effective tool for positively influencing the trajectories of both burnout and engagement. However, this study examined the effects of coaching on primary care physicians specifically, limiting the generalizability of the findings to leaders from other industry sectors. Additionally, the study used a combined score for examining burnout which is not only unadvisable by the founders of the Maslach Burnout Inventory scale (Maslach et al., 1996), but also does not provide insight on how each component of burnout may have been affected by the coaching intervention. Likewise, engagement was also not assessed by component, but rather one total score. Therefore, it is evident more coaching intervention research is needed to adequately address leadership burnout and work engagement.

Provided the strong negative correlation between burnout and engagement (Cole et al., 2012; Nimon and Shuck, 2020), we predict that an executive coaching intervention will decrease levels of emotional exhaustion, cynicism, and personal inefficacy and increase levels of dedication, vigor, and absorption for leaders at both within- and between-person levels. Finally, we expect the levels of these three dimensions of burnout and three dimensions of engagement in the control group to remain relatively steady.

*H1*: The executive coaching procedure will decrease the leader’s level of emotional exhaustion while the control group’s level of emotional exhaustion will remain stable.

*H2*: The executive coaching will decrease the leader’s level of cynicism while the control group’s level of cynicism will remain stable.

*H3*: The executive coaching will decrease the leader’s level of personal inefficacy while the control group’s level of personal inefficacy will remain stable.

*H4*: The executive coaching group will increase the leader’s level of vigor while the control group’s level of vigor will remain stable.

*H5*: The executive coaching group will increase the leader’s level of dedication while the control group’s level of dedication will remain stable.

*H6*: The executive coaching group will increase the leader’s level of absorption while the control group’s level of absorption will remain stable.

## 3 Materials and methods

### 3.1 Sample

In the initial phase, a one-month recruitment period took place between January and February of 2022. Here, clients of a human resources consultancy in Spain were invited by email to participate in the study. All included participants held a managerial position and agreed to complete a questionnaire at the start of the study protocol and again at the end (i.e., 10 weeks later). Thus, recruitment produced 100 managers from companies located in Spain. The managers were then randomly assigned to an experimental and control group, so that each group consisted of 50 managers. Eight participants (i.e., two from the experimental group and six from the control group) did not complete the survey at T2 and thus were omitted from the study. Thus, the final participant sample (see Table 1) included managers from Spain (*N =* 92; 64% male) ages 22 to 66 years of age (*M_age_* = 42.70, SD = 9.21). Referencing the Spanish education system, managers were primarily university educated (70%), though some had stopped after secondary education (23%) and even fewer after basic education (7%). Additionally, one manager did not indicate their educational level.

**Table 1:** Sociodemographic characteristics of participants at baseline for sample groupings.

Baseline Characteristic	Intervention group		Wait-list control group		Full sample	
	*n*	%	*n*	*%*	*N*	*%*
Gender						
Female	11	23.40	22	48.89	33	35.87
Male	36	76.60	23	51.11	59	64.13
Education^a^						
University	31	67.40	33	73.33	64	70.33
Secondary	10	21.74	11	24.44	21	23.08
Primary	5	10.87	1	2.22	6	6.59

*N* = 92 (intervention *n* = 47; control *n* = 45). Participants in the intervention group were on average 44 years old (SD = 10.29) and 41 years old (SD = 7.78) on average in the control group. ^a^One participant from the intervention group did not indicate their level of education.

### 3.2 Procedure

Research has revealed that interventions lasting between one and two months had the greatest influence on symptoms of burnout (Maricuţoiu et al., 2016). Therefore, the intervention period in our study was conducted over a ten-week period. Data from this longitudinal study were collected from participants by way of a questionnaire prior to the start of the intervention period (T1) and then again ten weeks later at the end (T2). Participants in the experimental group participated in the intervention where they attended ten executive coaching sessions once a week for approximately one hour. A total of seven experienced coaches were responsible for performing the executive coaching sessions. To ensure adequate training for this specific process of executive coaching, a manual was developed that describes in detail all the steps to follow in each of the 10 coaching sessions. In addition, each of the seven coaches received the coaching intervention, by the senior coaches of the human resources consultancy, prior to starting the process with the study participants. The senior coaches also met once a week with all the coaches for a training session to ensure the consistency of the intervention. Coaching sessions were conducted mostly face-to-face and online only in instances where it was impossible for the leader to attend a face-to-face session. The control group did not attend any coaching sessions, but were offered sessions after they completed the second questionnaire at T2.

#### 3.2.1 Executive coaching intervention

The coaching procedure used was a new tool for personal and professional development of managers. Relevant objectives of the coaching program for this study included reducing the stress and burnout levels of managers and increasing the levels of motivation, commitment, and performance of the managers and their work team. The first session included a reflection period where the coachee describes their life as well as what they perceive to be their main professional achievements. The session concluded with the coachee identifying specific achievable objectives that can be measured and are time bound. The second session, also began with a reflection period, but here only consisting of any pertinent events that took place in the past week (i.e., pleasant/unpleasant events, specific competencies used, and any weaknesses observed prior to unpleasant events). Next, coachees were guided by the coach to use the “Values and Anti-Values Tool.” Here, the tool had the coachee think about past or present figures and identify the characteristics they value and do not value. Then the coachee examined the resulting values and anti-values and identified which matched their own value system. The coachee was then asked to reflect on questions regarding their ideal vision of the world, their life’s mission, their mission as a leader, and their main work-related problems. Finally, the second session ended with a review of the coachee’s previously set objectives. Next, the coach invited the coachee to reflect on what they did to work toward these objectives in the past week and what they will do to work toward them further in the week to come. Here, the coach ensured to highlight any successes mentioned and encourage reflecting on possibilities to overcome any obstacles. The reflection of set career goals and objectives was continued in each of the following sessions excluding the tenth session (i.e., the third session to the ninth session).

In the third session, the coachee was asked to discuss an important work-related topic of their choice in which the coach and coachee proceeded to analyze. Next, the coachee was prompted with two open questions about their future. Here, the coach used active listening, note taking, and the occasional question while the coachee answered. The session concluded with the use of a strengths, weaknesses, opportunities, and threats (SWOT) analysis regarding the coachee’s desired professional life over the course of the following ten years. The fourth session included the coachee completing their part of a 360-degree evaluation (e.g., a skills assessment typically completed by subordinates, peers, supervisors, and the individual of interest) about their managerial skills (e.g., leadership, communication, motivation, teamwork, and self-control). Next, the fifth session involved the coach providing the coachee with a summary of the 360-degree evaluation results. Here, strengths and weaknesses in each of the skills assessed are emphasized. In the sixth session that followed, the coachee was provided with a detailed report of the results from the 360-evaluation. Here, the report includes the comparison of scores for each item across questionnaires completed. Additionally, the report included the coach’s identification of the coachees main strengths and areas of improvement based on the score comparisons. Once the coachee had time to review the report, the coach invited the coachee to develop an action plan in order address the skills needing improvement as identified in the report. Here, the coach asked the coachee how they plan to improve the identified skills and then proceeded to take notes and encourage the coachee to identify specific tasks for their action plan.

Session seven was centered around the coachee describing a detailed account of one day at work. The account was provided by the coachee via prompts by the coach (e.g., *“What do you do?,” “Who do you associate with?,” “How do you feel?,” “What would you change?,” “What are you most passionate about or what do you like the most?,”* and *“What do you like the least and what can you not stand?”*). Finally, the coachee reflected the aspects they do not like in their current role and how they could change them. Next, in the eighth session, the coachee identifies and then examines concerns that are resulting in “sleepless nights” and how they may mitigate the effects of the concerns. The identification and examination processes is facilitated by the coach by use of prompting questions (e.g., *“Who or who is involved in the problem?,” “Do you have ability to solve the problem yourself?,” “Is it urgent?,” “How much does it impact your state or mood?,”* and *“Indicate an action that you can carry out to reduce its effects.”*). In the ninth session, the coach focused on the coachee’s relation to their team. Here, the coachee identifies the members of their team to which they work with on a regular basis. Then, the coachee reflects on any areas of improvement as well as any strengths they have regarding their team members. Next, the coach asks the coachee to state specific actions they can engage in to promote team effectiveness. Finally, in the tenth session the coach provides a synthesis of the outputs of all prior sessions and a final action plan toward the coachee’s development of their leadership performance is produced.

### 3.3 Measures

#### 3.3.1 Burnout

Burnout was assessed using the Spanish version (Salanova et al., 2000) of the Maslach-Burnout Inventory-General Survey (MBI-GS) (Schaufeli et al., 1996). The 15-item survey assessed all three dimensions of burnout (i.e., exhaustion, cynicism, and professional inefficacy). Each item had participants respond on a 6-point Likert scale with “Never” at 1 and “Every day” at 6.

##### 3.3.1.1 Emotional exhaustion

Emotional exhaustion was assessed on a subscale of five items. Sample items include: “*I feel emotionally drained from my work*,” “*I am tired when I get up in the morning and have to face another day at my job*,” and “*Working all day is a strain for me*.” The average level of exhaustion was computed based on the five items. The Cronbach’s alpha coefficients were both 0.85 at T1 and at T2.

##### 3.3.1.2 Cynicism

Cynicism was assessed on a subscale of four items. Sample items include: “*I have lost interest in my work since I started in this position*,” “*I have lost enthusiasm for my work*,” and “*I doubt the significance and value of my work*.” The average level of cynicism was computed based on the four items. The Cronbach’s alpha coefficient at T1 was 0.78 and 0.80 at T2.

##### 3.3.1.3 Personal inefficacy

Professional inefficacy was assessed on a subscale of six items Sample items include: “*I can effectively solve problems that arise in my work*,” “*I contribute effectively to what my organization does*,” and “*In my opinion I am good at my job*.” The average level of professional inefficacy was computed based on the six items. The Cronbach’s alpha coefficient at T1 was 0.75 and 0.83 at T2.

#### 3.3.2 Engagement

Engagement was assessed with a 15 item engagement questionnaire adapted by Salanova et al. (2000) from the employee version of Schaufeli et al.’s (2002) engagement questionnaire. Furthermore, all three dimensions of engagement (i.e., vigor, dedication, and absorption) were assessed on 5-point Likert scale from “Never” at 1 and “Always” at 5.

##### 3.3.2.1 Vigor

Vigor was assessed on a scale of six items. Sample items include: *“At my work, I feel bursting with energy,” “At my work I always persevere, even when things do not go well,”* and *“At my job, I am very resilient, mentally.”* The average level of vigor was computed based on the six items. The Cronbach’s alpha coefficient at T1 was 0.77 and 0.80 at T2.

##### 3.3.2.2 Dedication

Dedication was assessed on a scale of five items. Sample items include: *“To me, my job is challenging,” “My job inspires me*,” and *“I am enthusiastic about my job.”* The average level of dedication was computed based on the five items. The Cronbach’s alpha coefficients were 0.86 at T1 and T2.

##### 3.3.2.3 Absorption

Absorption was assessed on a scale of six items. Sample items include: “*When I am working, I forget everything else around me*,” “*Time flies when I am working*,” and “*It is difficult to detach myself from my job*.” The average level of absorption was computed based on the six items. The Cronbach’s alpha coefficient at T1 was 0.84 and 0.87 at T2.

### 3.4 Analysis

All analyses were conducted using SPSS 28 software (IBM Corp, 2021). A preliminary analysis assessed whether leaders in the experimental group and control group differed in terms of demographic variables (i.e., age, gender, and education). An independent samples *t*-test explored any difference in the average age between groups and *χ*^2^ tests examined any differences in gender or education between groups. Additionally, independent samples *t*-tests assessed whether the outcome variables (i.e., emotional exhaustion, cynicism, personal inefficacy, vigor, dedication, and absorption) at T1 differed among leaders between groups. To test the effectiveness of the executive coaching intervention, we conducted repeated measures analyses of variance (ANOVAs) where time (i.e., T1 vs. T2 for each dimension for burnout and engagement respectively) was our independent within-subject factor and intervention groups (e.g., experimental and control) were our independent between-subjects factor. In the event of a significant interaction effect between time and group, follow-up analyses with paired samples *t*-tests determined whether the means from groups differed significantly between T1 and T2 on the respective outcome variables. We considered *p* ≤ 0.05 to be statistically significant. Practical significance was determined by effect size using Cohen’s *d_s_*, which was interpreted as 0.20 small, 0.50 medium, and 0.80 large (Cohen, 1988).

## 4 Results

### 4.1 Preliminary analysis

An independent samples *t*-test revealed that there was not a significant difference in age between the experimental and control group [*t*(85) = 1.544, *p* = 0.13]. Additionally, the *χ*^2^ tests revealed that leader’s education level did not differ between groups [*χ*^2^(2) = 2.77, *p* = 0.25], but the groups did significantly differ in gender [*χ*^2^(1) = 6.50, *p* = 0.01] (see Table 1). However, a regression analysis showed that gender did not significantly impact any of the T1 outcome variables (i.e., emotional exhaustion [*F*(1,89) = 1.09, *p* = 0.30], cynicism [*F*(1,89) = 1.62, *p* = 0.21], personal inefficacy [*F*(1,89) = 0.23, *p* = 0.63], vigor [*F*(1,89) = 0.01, *p* = 0.92], dedication [*F*(1,89) = 0.13, *p* = 0.72], absorption [*F*(1,89) = 0.15, *p* = 0.70]). Thus, age, education and gender were excluded from further analyses. Levene’s test of equality of variances revealed that both the experimental group and control group had equal variances at both T1 and T2 for emotional exhaustion, personal inefficacy, vigor, dedication, and absorption. However, the assumption of equal variances was violated with respect to cynicism at T2 [*F*(1,89) = 0.64, *p* < 0.05]. Therefore, caution should be exercised when interpreting any main effects or interactions including this T2 outcome variable.

### 4.2 Hypotheses test results

Repeated measures ANOVAs assessed all six of our hypotheses sequentially as outlined in the introduction of this paper (see Table 2).

**Table 2:** Means, standard errors (in brackets) of the outcome variables as a function of time (T1 and T2) and group (intervention and control).

Variable	Intervention (*n =* 47)		Wait-list control (*n =* 45)		Time	Group	Time × group
	*T1*	*T2*	*T1*	*T2*			
Burnout^a^							
Emotional Exhaustion	2.54 (0.91)	2.31 (0.57)	2.31 (0.73)	2.31 (0.72)	*F*(1,89) = 3.78* η^2^*_G_* = 0.01	*F*(1,89) = 0.68	*F*(1,89) = 3.78* η^2^*_G_* = 0.01
Cynicism	1.73 (0.58)	1.54 (0.47)	1.57 (0.65)	1.64 (0.60)	*F*(1,89) = 0.80	*F*(1,89) = 0.09	*F*(1,89) = 4.29* η^2^*_G_* = 0.01
Personal Inefficacy	4.20 (0.41)	4.30 (0.37)	4.29 (0.47)	4.16 (0.49)	*F*(1,89) = 0.28	*F*(1,89) = 0.10	*F*(1,89) = 8.58** η^2^*_G_* = 0.02
Engagement							
Vigor	3.77 (0.79)	3.93 (0.66)	4.00 (0.67)	3.88 (0.70)	*F*(1,90) = 0.09	*F*(1,90) = 0.38	*F*(1,90) = 4.70* η^2^*_G_* = 0.01
Dedication	3.96 (0.85)	3.90 (0.70)	4.07 (0.78)	4.02 (0.83)	*F*(1,90) = 0.64	*F*(1,90) = 0.62	*F*(1,90) = 0.01
Absorption	3.58 (0.93)	3.57 (0.85)	3.51 (0.87)	3.60 (0.84)	*F*(1,89) = 0.22	*F*(1,89) = 0.01	*F*(1,89) = 0.50

Results are based on two assessments (*N* = 92). *p ≤ 0.05. **p ≤ 0.01. ^a^Control group scores from a smaller sample (*n* = 44) as one participant did not complete this measure.

**Figure 1:**
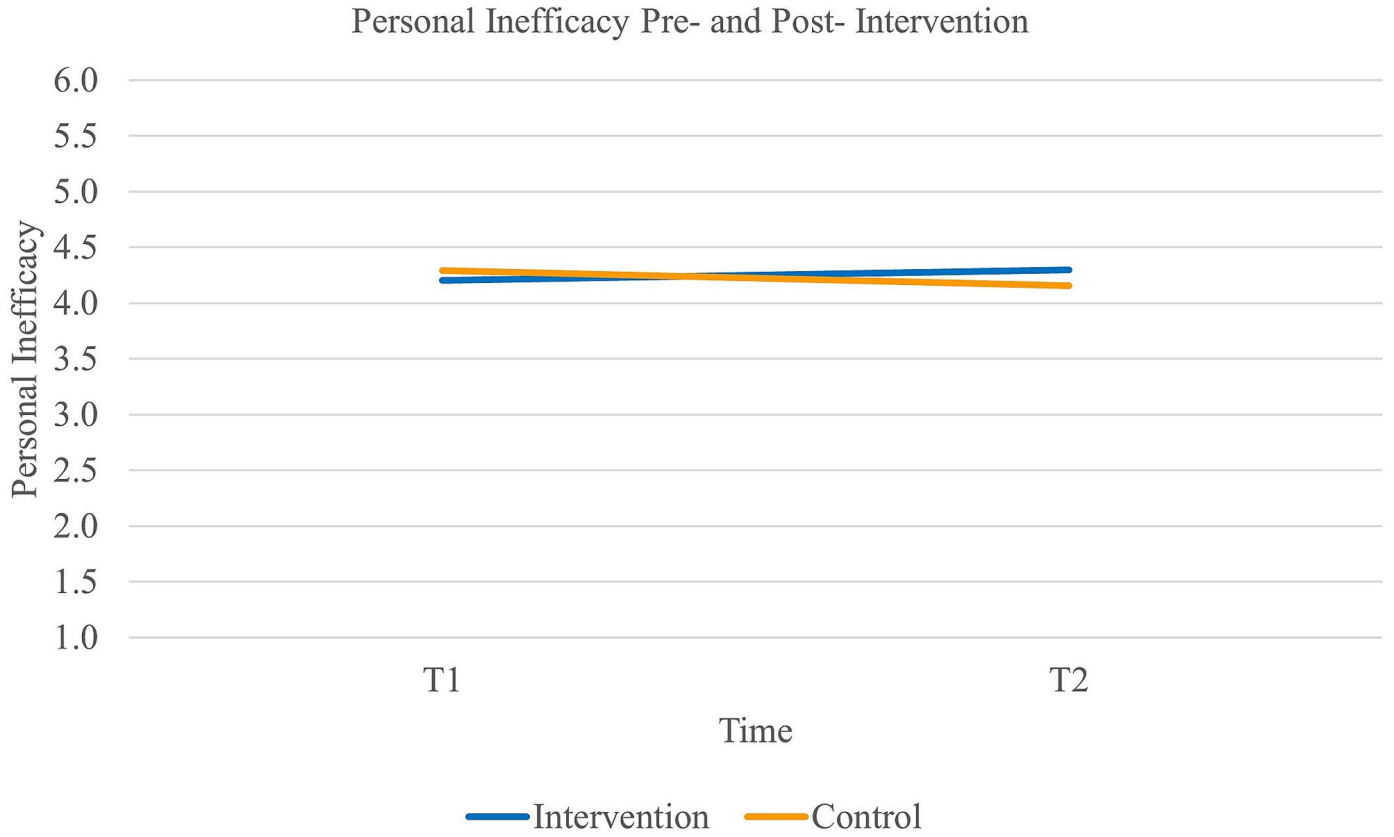
Interaction between time and group on the levels of emotional exhaustion.

First, results revealed a main effect of time on *emotional exhaustion* [Wilk’s Lambda = 0.96, *F*(1,89) = 3.78, *p* = 0.05, η^2^*_p_* = 0.04, η^2^*_G_* = 0.01], but no effect of group [*F*(1,89) = 0.68, *p* = 0.41]. However, there was a significant interaction effect of time and group on emotional exhaustion [Wilk’s Lambda = 0.96, *F*(1,89) = 3.78, *p* = 0.05, η^2^*_p_* = 0.04, η^2^*_G_* = 0.01]. Paired samples *t*-tests further revealed that the experimental group significantly decreased in their level of emotional exhaustion (*t*(46) = 2.41, *p* = 0.01, 95% CI [0.04, 0.43]) while the control croup remained stable [*t*(43) = 0.00, *p* = 0.50] (see Figure 1). Additionally, the effect size of the experimental group was medium to large (Cohen’s *d_s_* = 0.67). Thus, our first hypothesis was supported.

**Figure 2:**
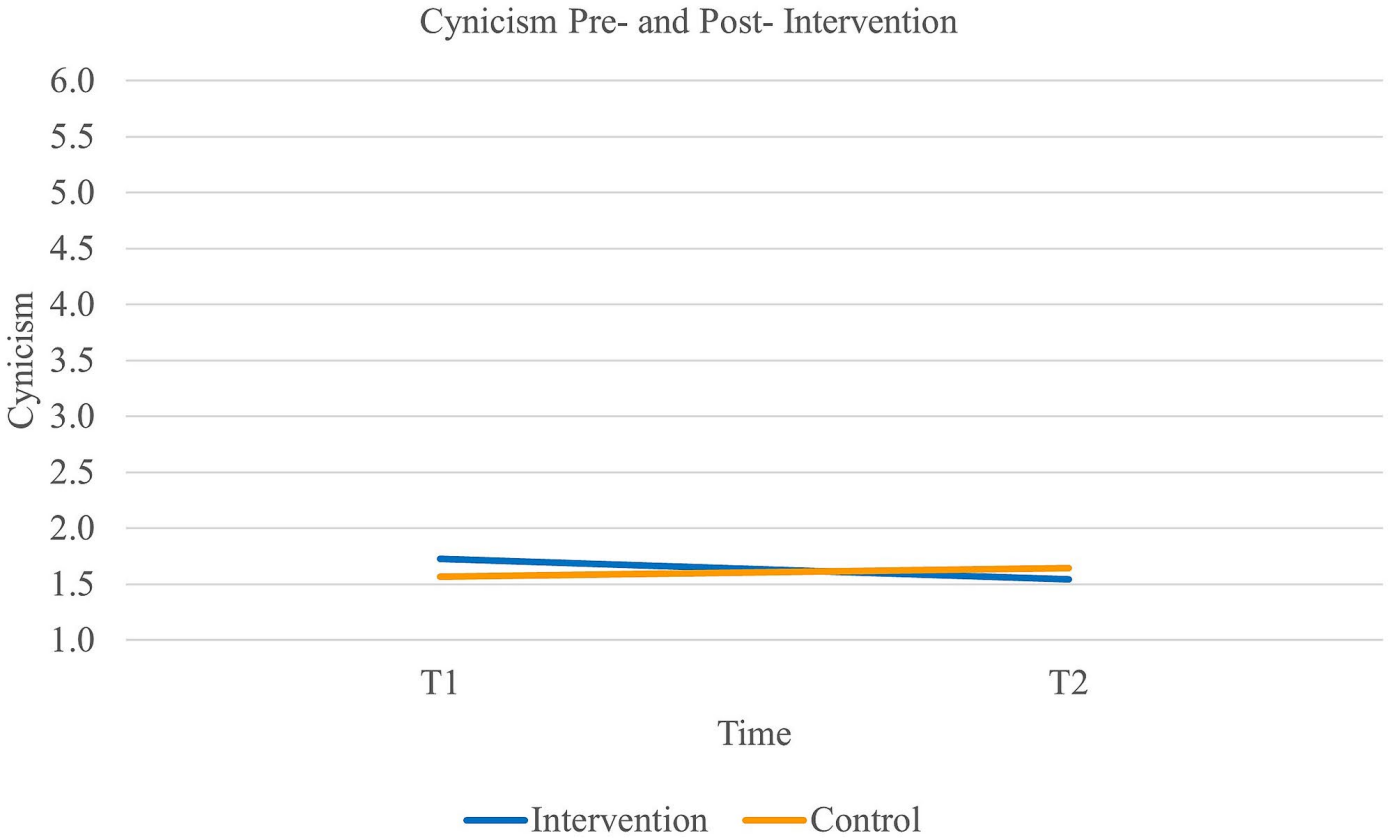
Interaction between time and group on the levels of cynicism.

Second, results did not reveal a main effect of time on *cynicism* [Wilk’s Lambda = 0.99, *F*(1,89) = 0.80, *p* = 0.37] or group [*F*(1,89) = 0.09, *p* = 0.77]. However, there was a significant interaction effect of time and group on cynicism [Wilk’s Lambda = 0.95, *F*(1,89) = 4.29, *p* < 0.05, η^2^*_p_* = 0.05, η^2^*_G_* = 0.01]. Paired samples *t*-tests further revealed that the experimental group significantly decreased in their level of cynicism (*t*(46) = 1.85, *p* < 0.05, 95% CI [−0.02, 0.39]) while the control group remained stable [*t*(43) = −1.01, *p* = 0.16] (see Figure 2). Additionally, the effect size of the experimental group was medium to large (Cohen’s *d_s_* = 0.69). Thus, our second hypothesis was supported.

**Figure 3:**
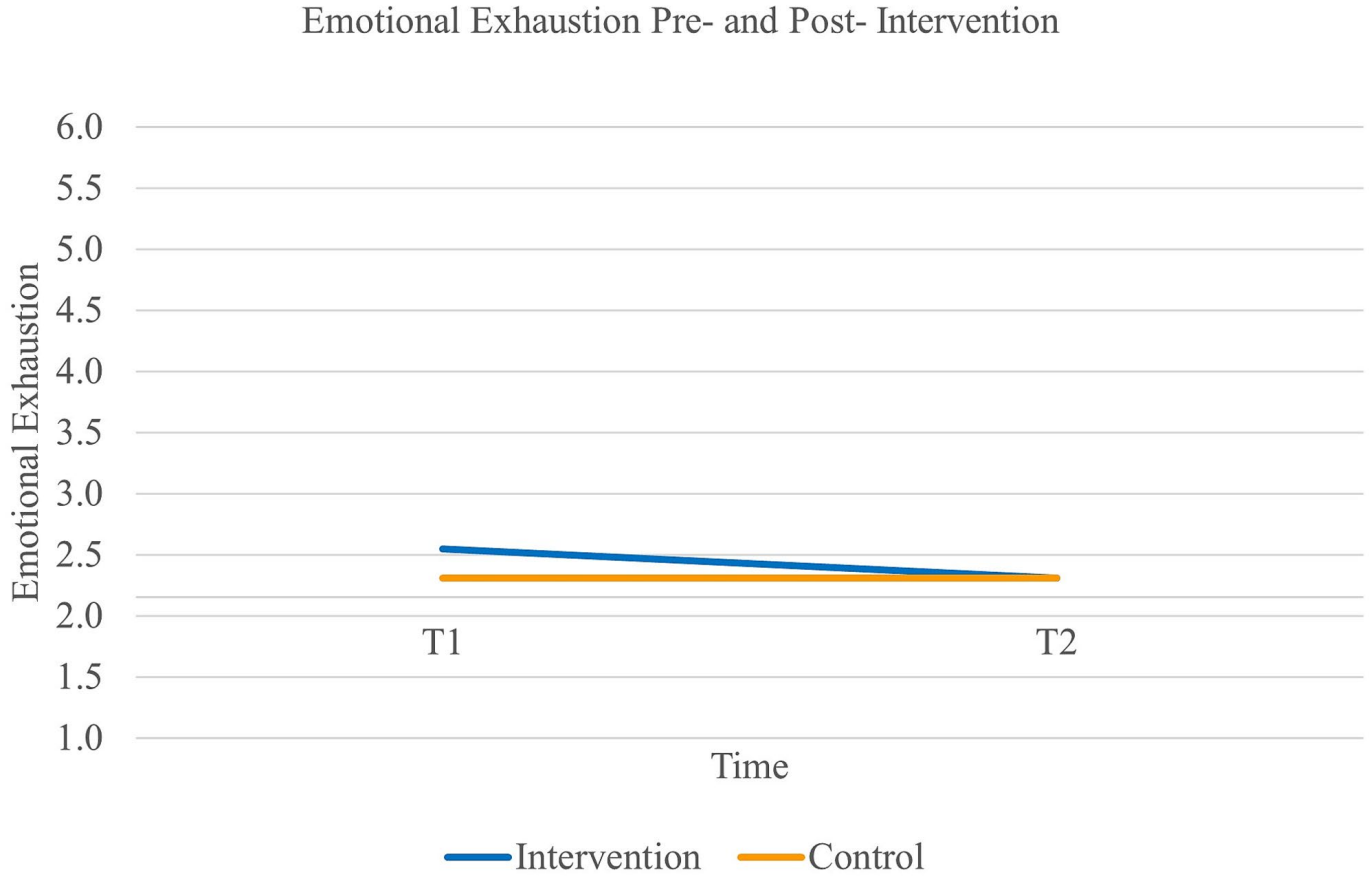
Interaction between time and group on the levels of personal inefficacy.

Third, results did not reveal a main effect of time on *personal inefficacy* [Wilk’s Lambda = 1.00, *F*(1,89) = 0.28, *p* = 0.60] or group [*F*(1,89) = 0.10, *p* = 0.75]. However, there was a significant interaction effect of time and group on personal inefficacy [Wilk’s Lambda = 0.91, *F*(1,89) = 8.58, *p* < 0.01, *η*^2^*_p_* = 0.09, η^2^*_G_* = 0.02]. Paired samples *t*-tests further revealed that the experimental group significantly decreased in their level of personal inefficacy (*t*(46) = −1.81, *p* < 0.05, 95% CI [−0.19, 0.1]) with a medium to large effect size (Cohen’s *d_s_* = 0.69) (see Figure 3). Additionally, the control group increased in their level of personal inefficacy (*t*(43) = 2.30, *p* = 0.01, 95% CI [0.02, 0.25]) with a small to medium effect size (Cohen’s *d_s_* = 0.38). Thus, our third hypothesis was partially supported.

**Figure 4:**
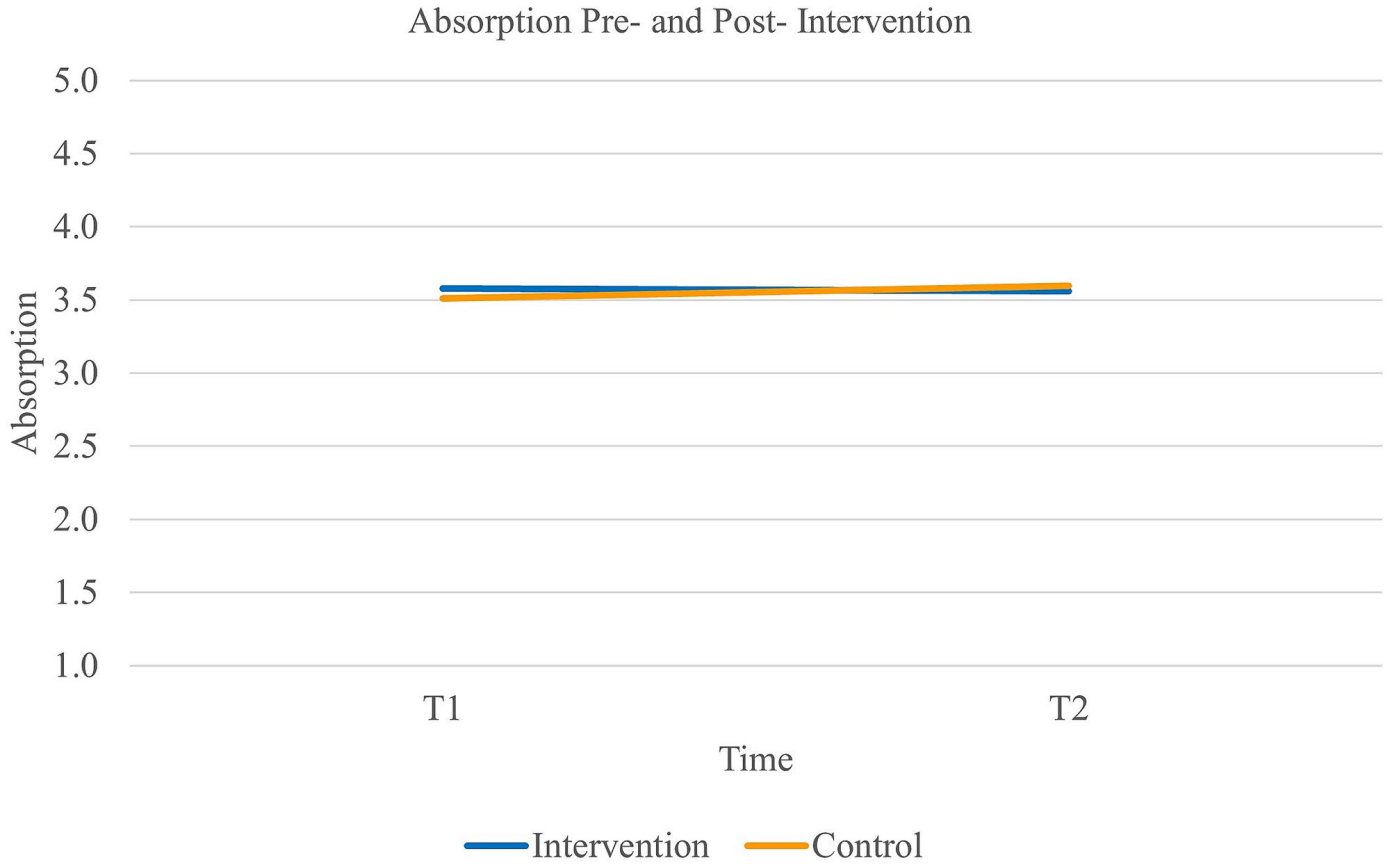
Interaction between time and group on the levels of vigor.

Fourth, results did not reveal a main effect of time on *vigor* [Wilk’s Lambda = 1.00, *F*(1,90) = 0.09, *p* = 0.77] or group [*F*(1,90) = 0.38, *p* = 0.54]. However, there was a significant interaction effect of time and group on vigor [Wilk’s Lambda = 0.95, *F*(1,90) = 4.70, *p* < 0.05, *η*^2^*_p_* = 0.05, η^2^*_G_* = 0.01]. Paired samples *t*-tests further revealed that the experimental group significantly increased in their level of vigor (*t*(46) = −1.66, *p* = 0.05, 95% CI [−0.35, 0.03]) while the control group remained stable [*t*(44) = 1.41, *p* = 0.08] (see Figure 4). Additionally, the effect size of the experimental group was medium to large (Cohen’s *d_s_* = 0.65). Thus, our fourth hypothesis (H_4_) was supported.

**Figure 5:**
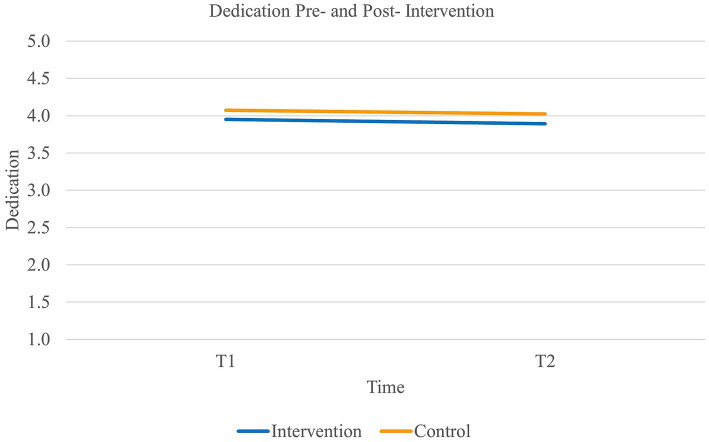
Interaction between time and group on the levels of dedication.

**Figure 6:**
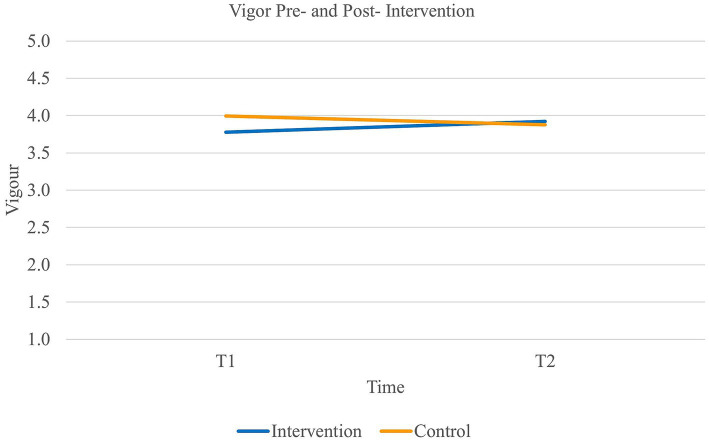
Interaction between time and group ion the levels of absorption.

Finally, results did not reveal a main effect of time on *dedication* [Wilk’s Lambda = 0.99, *F*(1,90) = 0.64, *p* = 0.43] or on *absorption* [Wilk’s Lambda = 1.00, *F*(1,89) = 0.22, *p* = 0.64]. Neither did the results reveal an effect of group on dedication [*F*(1,90) = 0.62, *p* = 0.43] or absorption *F*(1,89) = 0.01, *p* = 0.92). Finally, there did not appear to be a significant interaction effect of time and group on either dedication [Wilk’s Lambda =1.00, *F*(1,90) = 0.01, *p* = 0.94] or absorption (Wilk’s Lambda = 0.99, *F*(1,89) = 0.50, *p* = 0.48) either (see Figures 5, 6). Thus, our fifth and sixth hypotheses were not supported.

## 5 Discussion

To help fill the gaps in the literature regarding a lack of longitudinal and experimental designs for examining outcomes of executive coaching procedures, we conducted a randomized control trial study where data was collected at two time points to examine how an executive coaching procedure may positively influence the levels of burnout and engagement in leaders. Using repeated measures ANOVAs, we examined if there were associations between the executive coaching intervention and the amount of burnout and engagement the leader experienced over time. Consistent with previous coaching literature suggesting that lower levels of burnout are associated with a coaching intervention (Grant, 2017; Edú-Valsania et al., 2022), the present study results suggest that leaders who take part in executing coaching sessions can significantly reduce their amount of burnout. Further, leaders who took part in the executive coaching intervention reported less emotional exhaustion, cynicism, and personal inefficacy after attending the 10 coaching sessions. Thus, each dimension of burnout was significantly reduced. Additionally, the moderate effect sizes of reduced burnout in the intervention group are well in line with previous randomized control trial studies examining coaching (de Haan and Nilsson, 2023). Thus, providing a more appropriate interpretation of the effect sizes found in this study versus a comparison to Cohen’s (1988) prescribed general guidelines for effect size (Lakens, 2013).

Additionally, our hypothesis that the leaders who did not undergo the intervention would maintain relatively stable levels of emotional exhaustion, cynicism, and personal inefficacy was partially supported. While the leaders in the control group did maintain stable levels of emotional exhaustion and cynicism throughout the study protocol, their levels of personal inefficacy appeared to increase over time. Thus, we found partial support for our third hypothesis. The instability of the personal inefficacy levels reported by the control group could be related to some of the recent discourse about the inclusion of personal inefficacy as a dimension of burnout (Schaufeli et al., 2020; Haar, 2021). Considering that personal inefficacy is closely related to low self-efficacy (Shoji et al., 2016) some researchers have proposed that personal inefficacy is a consequence of burnout rather than a dimension of the concept (Schaufeli et al., 2020). Therefore, it may be that the control group increased in their level of personal inefficacy over time due to untreated burnout symptoms.

Furthermore, of our three hypotheses expecting engagement to increase for the intervention group, only our fourth hypothesis was supported. Indeed, vigor was the only dimension of engagement that increased for leaders in the executive coaching intervention while the control group remained stable. One possibility for the elevated levels of vigor reported by the leaders from intervention group could be due to the use of feedback tools such as 360-degree feedback. Feedback is generally perceived as extremely beneficial for the coachee and feedback alone has been shown to have comparable effects to that of a full coaching procedure (Smither, 2011; Pandolfi, 2020). Such coachee benefits for leaders can include the perception that their organization values them and in turn leaders may feel more invested in their occupation (Schwartz et al., 2022). Therefore, the feedback aspect of the coaching procedure may have aided the coachees in experiencing more vigor in their work. Further, our coaching procedure included coachees reflecting on their work experiences by way of a SWOT analysis. Here, when coachees reflected on their positive experiences revealed through the analysis they worked on ways in which to repeat or maintain these experiences in the future. Thus, this reflective process can allow for the coachee to become aware of their beneficial prospects which may prompt engagement (McGonagle et al., 2020). However, our study did not find a significant interaction effect of intervention and time for dedication and absorption. In other words, leaders from the executive coaching group did not improve in their level of dedication and absorption. However, the lack of suggested impact of the intervention does appear to be consistent with prior studies that have sought to use interventions to increase engagement levels in workers (Knight et al., 2019). Indeed, in Knight et al.’s (2019) systematic review of engagement interventions for employees, the researchers found that it was more likely for interventions to reveal positive significant results for vigor than for dedication or absorption. Further, the researchers also found that studies where participants reported lower average work engagement scores at T1 found that the given engagement intervention significantly increased the worker’s level of engagement. Indeed, both the intervention and control group in our study reported relatively high scores for dedication and absorption. Therefore, it could be that the executive coaching intervention was not able to increase these dimensions even more than what was already present in the leaders. Moreover, it has also been argued that individuals with lower levels of dedication and absorption benefit more from an intervention aimed at increasing their levels of engagement because they have more to gain (Ouweneel et al., 2013). For example, prior research found individuals with higher levels of exhaustion or lower levels of work engagement were able to increase their engagement levels after taking part in an intervention targeting work engagement (Vuori et al., 2012; Ouweneel et al., 2013). Indeed, in addition to the sample in the present study having higher levels of dedication and absorption at T1, the leaders also reported low levels emotional exhaustion, cynicism, and personal inefficacy at T1. Thus, perhaps the executive coaching procedure may be more efficient for increasing dedication and absorption for leaders who have higher levels of burnout and lower levels of engagement.

### 5.1 Implications

The findings from this study reveal that use of an executive coaching procedure can reduce burnout symptoms in leaders. Provided that so many leaders are being affected by burnout, organizations should look to implement coaching programs for the leaders as a form of risk management. Further, leaders should take it upon themselves to partake in executive coaching sessions to improve their energy levels toward their work and their ability to persevere when confronted with workplace challenges. Moreover, as this study did not find that all components of engagement can increase from executive coaching for leaders with relatively high levels of engagement, organizations may benefit more from implementing an executive coaching procedure when their leaders have lower levels of engagement.

### 5.2 Strengths, limitations, and future directions

The present study contributes to the limited executive coaching research using longitudinal randomized control designs to examine any coaching outcomes. Thus, the results of this study allow for a more meaningful understanding of the effects of an executive coaching intervention over time. However, we believe that future studies should consider including a follow-up assessment after the post-test questionnaire. A third time point would enable interpretation of a linear trend in the leader’s engagement and burnout levels which would allow for a richer understanding of the executive coaching effects. For example, a follow up assessment would help to capture whether dedication and absorption simply need more time to develop or not. Similarly, daily assessments by way of an ecological momentary assessment would also help to determine linear trends in engagement and burnout. Indeed, previous research has found that burnout levels may fluctuate daily (Bakker et al., 2014). Further, engagement levels also appear to fluctuate daily and these within-person fluctuations may make up almost half of the total variance in daily work engagement (Bakker, 2014). Thus, an ecological momentary assessment design could provide more context around the levels of burnout and engagement in leaders. Additionally, while this study benefited from having a diverse variety of leaders in terms of sector, organization, and position, we did not collect standardized data on any of these variables. Consequently, a recent study by Haar (2021) revealed that a leader’s status (e.g., senior managers versus a CEO) may influence the amount of burnout a leader experiences. Thus, future studies may want to examine how executive coaching may influence burnout and engagement in leaders based on their level within the organization. Similarly, consideration of other pre-existing variables could be beneficial. For example, unlike other organizational interventions, executive coaching may require some antecedent qualities in the coachee for overall coaching success. For example, the effectiveness of the coaching intervention can be impacted by the coachee’s motivation, self-efficacy, and learning goal orientation (Bozer and Jones, 2018). However, despite these precursing factors for effectiveness, the results of our study indicate that executive coaching can still have a positive influence on leaders. Though, future studies could examine how the levels of these antecedent factors may influence the outcome of this executive coaching procedure for the coachee.

Moving on, the present study included some limitations regarding generalizability of the results. First, all participants in our study volunteered to participate. This is a particular strength for a coaching intervention study as a key factor in the success of a coaching procedure is the coachee’s willingness to change. Thus, through voluntarily signing up for the study the leaders indicated a desire to make positive behavior changes. However, it would be important to explore how a leader’s openness to change may impact the outcomes of an executive coaching procedure and thus allowing for more generalizability. In the same way, evaluating potential factors related to the intervention process would likely facilitate greater understanding of how effective the executive coaching procedure may be for improving burnout and decreasing engagement in leaders (Nielsen et al., 2007; Lai and Palmer, 2019). For example, future studies could evaluate not only the leader’s willingness to change (i.e., as a contextual aspect of the intervention process), but also other relevant process variables related to the context and setting of the intervention (e.g., the coach and coachee’s characteristics and behaviors, as well as components of the coach-coachee relationship), how the intervention is implemented, and the coach and coachee’s perceptions of the intervention (Nielsen et al., 2007; Pandolfi, 2020). Considering that that the components of the executive coaching process can significantly impact the success of the intervention (Lai and Palmer, 2019) and that there is a lack of studies that have examined the executive coaching process (Pandolfi, 2020), use of a process evaluation in future studies is highly advisable.

Second, the executive coaching procedure used the GROW model, which was appropriate as the model is well suited for Western-cultures (Palmer and Whybrow, 2019), however not necessarily for other parts of the world. Therefore, use of the GROW model hinders the generalizability of the study’s results to other cultures. Third, the sample from this study consistent of mostly male participants. This could be expected given that there still remains a gender gap favoring males in terms of global entrepreneurship (Global Entrepreneurship Monitor, 2023). Additionally, in Spain there are more male established business owners than women. However, while we did not find any significant influence of gender on the results from this study, we believe that future research should strive for a more balanced sample where possible. Finally, the present study had some statistical limitations. First, a smaller sample size (*N* = 92) limited our statistical power, thus study replication with a larger sample would aid the interpretation of the results. Additionally, as mentioned, the effect sizes found for the intervention group’s level of emotional exhaustion, cynicism, personal inefficacy, and vigor were deemed moderate. However, caution should be used when interpreting these effect sizes given that self-reports tend to inflate the effect size compared to objective measures (Athanasopoulou and Dopson, 2018; de Haan and Nilsson, 2023).

## 6 Conclusion

The yearly increase of leaders becoming burnt-out is a point of concern not only for the well-being of the leaders, but also for maintaining the performance of organizations they lead, as well as the well-being of the employees within those organizations. Thus, ways in which to reduce burnout symptoms in leaders should be sought. Our study explored how an executive coaching procedure may reduce burnout in leaders while concurrently increasing their work engagement. Results revealed that leaders who took part of the executive coaching intervention had less emotional exhaustion, cynicism, and personal inefficacy, as well as increased levels of vigor by the end of the intervention period. Thus, our study suggests that executive coaching can be a useful risk management tool to mitigate the potential negative outcomes from leaders who burnout.

## Data availability statement

The raw data supporting the conclusions of this article will be made available by the authors, without undue reservation.

## Ethics statement

The studies involving humans were approved by the Universidad de Valencia which is affiliated with the Institut d’Investigació en Psicologia dels Recursos Humans, del Desenvolupament Organitzacional, i de la Qualitat de Vida Laboral (Idocal). The studies were conducted in accordance with the local legislation and institutional requirements. The participants provided their written informed consent to participate in this study.

## Author contributions

PB: Formal analysis, Visualization, Writing – original draft, Writing – review & editing. PR: Conceptualization, Data curation, Methodology, Project administration, Resources, Supervision, Writing – review & editing. CS: Conceptualization, Investigation, Project administration, Resources, Writing – review & editing. MT: Conceptualization, Investigation, Project administration, Resources, Writing – review & editing.
