# Supplementary material for: Coaching leaders toward favorable trajectories of burnout and engagement
Source: Front Psychol. 2023 Dec 4;14:1259672. doi: 10.3389/fpsyg.2023.1259672 (PMC10726131; doi:10.3389/fpsyg.2023.1259672)
Supplement: Supplementary file 1 [file Table_1.DOCX]

Supplementary Material

Table 3. Correlations among the dimensions of the study variables

|  | Correlation | | | | | | | | | | | |  |
| --- | --- | --- | --- | --- | --- | --- | --- | --- | --- | --- | --- | --- | --- |
| Variable | Emotional Exhaustion | | Cynicism | | Personal Inefficacy | | Vigor | | Dedication | | Absorption | | |
|  | T1 | T2 | T1 | T2 | T1 | T2 | T1 | T2 | T1 | T2 | T1 | T2 | |
| Emotional Exhaustion | — | — |  |  |  |  |  |  |  |  |  |  | |
| Cynicism | 0.55** | 0.38** | — | — |  |  |  |  |  |  |  |  | |
| Personal Inefficacy | -0.19 | -0.40** | -0.39** | -0.27** | — | — |  |  |  |  |  |  | |
| Vigor | -0.39** | -0.45** | -0.48** | -0.32** | 0.37** | 0.52** | — | — |  |  |  |  | |
| Dedication | -0.26* | -0.41** | -0.45** | -0.28** | 0.33** | 0.43** | 0.74** | 0.60** | — | — |  |  | |
| Absorption | -0.01 | -0.23* | -0.10 | -0.24* | 0.37 | 0.19 | 0.44* | 0.53** | 0.51** | 0.60** | — | — | |

*Note.* Results are based on the pre-test (T1) and post-test (T2) assessments (*N* = 92). **p* < .05. ***p* < .01.
